# Positional plasticity in regenerating *Amybstoma mexicanum* limbs is associated with cell proliferation and pathways of cellular differentiation

**DOI:** 10.1186/s12861-015-0095-4

**Published:** 2015-11-23

**Authors:** Catherine D. McCusker, Antony Athippozhy, Carlos Diaz-Castillo, Charless Fowlkes, David M. Gardiner, S. Randal Voss

**Affiliations:** Department of Biology, University of Massachusetts, Boston, MA 02125 USA; Department of Biology, Spinal Cord and Brain Injury Research Center, University of Kentucky, Lexington, KY 40506 USA; Department of Developmental and Cellular Biology, University of California, Irvine, CA 92602 USA; Donald Bren School of Information and Computer Science, University of California, Irvine, CA 92602 USA

**Keywords:** Limb regeneration, Positional information, Plasticity, Intercalation, Differentiation, Extracellular matrix, Microarray

## Abstract

**Background:**

The endogenous ability to dedifferentiate, re-pattern, and re-differentiate adult cells to repair or replace damaged or missing structures is exclusive to only a few tetrapod species. The Mexican axolotl is one example of these species, having the capacity to regenerate multiple adult structures including their limbs by generating a group of progenitor cells, known as the blastema, which acquire pattern and differentiate into the missing tissues. The formation of a limb regenerate is dependent on cells in the connective tissues that retain memory of their original position in the limb, and use this information to generate the pattern of the missing structure. Observations from recent and historic studies suggest that blastema cells vary in their potential to pattern distal structures during the regeneration process; some cells are plastic and can be reprogrammed to obtain new positional information while others are stable. Our previous studies showed that positional information has temporal and spatial components of variation; early bud (EB) and apical late bud (LB) blastema cells are plastic while basal-LB cells are stable. To identify the potential cellular and molecular basis of this variation, we compared these three cell populations using histological and transcriptional approaches.

**Results:**

Histologically, the basal-LB sample showed greater tissue organization than the EB and apical-LB samples. We also observed that cell proliferation was more abundant in EB and apical-LB tissue when compared to basal-LB and mature stump tissue. Lastly, we found that genes associated with cellular differentiation were expressed more highly in the basal-LB samples.

**Conclusions:**

Our results characterize histological and transcriptional differences between EB and apical-LB tissue compared to basal-LB tissue. Combined with our results from a previous study, we hypothesize that the stability of positional information is associated with tissue organization, cell proliferation, and pathways of cellular differentiation.

**Electronic supplementary material:**

The online version of this article (doi:10.1186/s12861-015-0095-4) contains supplementary material, which is available to authorized users.

## Background

Urodele amphibians such as salamanders and newts are exceptional model organisms to study processes of endogenous reprogramming and regeneration because they are capable of regenerating complicated biological structures from mature adult tissues. Regeneration in these organisms occurs through the modification of mature adult cells into regeneration-competent cells, known as blastema cells, which re-pattern and re-differentiate into the missing structures. One fascinating aspect of the regenerative process is that the regenerating blastema tissues only replace the structures that are missing, and thus a unique developmental pattern is established in the blastema depending on the location of the injured tissue. There are two main hypotheses to explain when and how this unique pattern is established in regenerating limbs, both of which have compelling supporting evidence. The first hypothesis is known as the “pre-specification model”, and is based on the idea that the pattern of the entire regenerate is established in the blastema as soon as it forms [1]. The second hypothesis, which has multiple sub-hypotheses, is broadly based on the idea that the pattern of the regenerate is generated gradually as the blastema develops [2, 3].

Recently published observations from our lab and others, suggest that patterning information is initially plastic and is gradually stabilized in specific regions of the blastema as it develops. One piece of evidence that strongly supports this hypothesis is that positional information in blastema cells can be reprogrammed when exposed to Retinoic Acid (RA) [4, 5]. For example, if an early bud (EB) stage blastema is exposed to RA, its positional information is reprogrammed such that it will form a complete limb regardless of the location of the blastema along the proximal/distal axis of the limb [4, 6]. A different reprogramming phenotype occurs if a late bud (LB) stage blastema is exposed to exogenous RA. In this situation, positional information of cells at the apical tip of the blastema is reprogrammed, while cells in the basal region closest to the stump are unaltered [4]. We attribute this difference in reprogramming capacity to reflect the stability of positional information in these tissues. We suspect that the state of tissue differentiation may be related to the stability of positional information in these cells because basal blastema cells are actively differentiating, while apical cells remain undifferentiated. One observation that supports this hypothesis is that positional information in uninjured limb tissues is unaffected by RA exposure [4, 5], despite the expression and activation of RA receptors in these cells [7, 8]. These observations reveal that an EB blastema and the apical tip of a LB blastema are capable of positional reprograming, and that differentiated cells are resistant to being reprogrammed.

While the above-described observations on RA-treated blastemas are consistent with the idea that EB and apical-LB cell populations are positionally plastic, we have further tested this hypothesis by determining whether positional reprogramming could occur through endogenous interactions between these and mature tissues at different positions along the limb [9]. Our experiments rest on the following observation – when cells with stabilized positional information are confronted with cells with differing positional information, the confronted cells induce the formation of new pattern (resulting in the formation of new structure) [10, 11]. Thus, if the confrontation of cells from different locations on the limb does not induce the formation of new structure, then one or both of the populations do not have stabilized positional information. Because mature limb tissues have stabile positional information [9, 12, 13], then the ability to induce the formation of new structure must reflect the stability of positional information in the “other” population of cells. Within this logical framework, we grafted EB and apical-LB tissues next to mature tissues at different locations along the limb. These confrontations did not induce the formation of new structures [9]. Since EB and apical-LB blastema tissues express region-specific Hox genes [1, 2], it is likely that they have positional information. Thus, the inability to induce new structure when grafted to a different position on the limb is probably due to plasticity (or instability) of positional information, rather than lack of this information. Also consistent with this idea is the observation that the expression of region-specific T-Box (*Tbx*) genes is altered in EB grafts such that they match the expression profile of the new host location [9].

As we stated above, uninjured limb tissue has stabile positional information, and thus can induce the formation of new structures when confronted with cells with differing positional information. We have found that the basal region of the LB blastema also has this inductive capacity, revealing that positional information is stabile in the basal region of a LB blastema. Thus, a LB blastema is composed of both positionally plastic (apical) and positionally stable (basal) populations of cells [9].

Understanding the nature of positional plasticity and the mechanisms that control this property is not only important for understanding pattern formation during limb regeneration, but also could help us improve the efficacy of regenerative therapies that attempt to engraft cells with positional information [14]. With these objectives in mind, we used histological and microarray analysis to identify cellular processes, genes, and signaling pathways that differentiate EB and apical-LB cell populations, from the basal-LB population. Relative to the EB and apical-LB populations, the basal-LB population showed greater actin cytoskeleton and ECM structural organization, and yielded higher expression estimates for genes that promote cellular differentiation. Combined with our previous results [9], we hypothesize that that positional plasticity may be associated with factors that regulate ECM organization and cellular differentiation.

## Results

### Characterization of tissue organization in EB and apical-LB compared to basal-LB populations

**Fig. 1:**
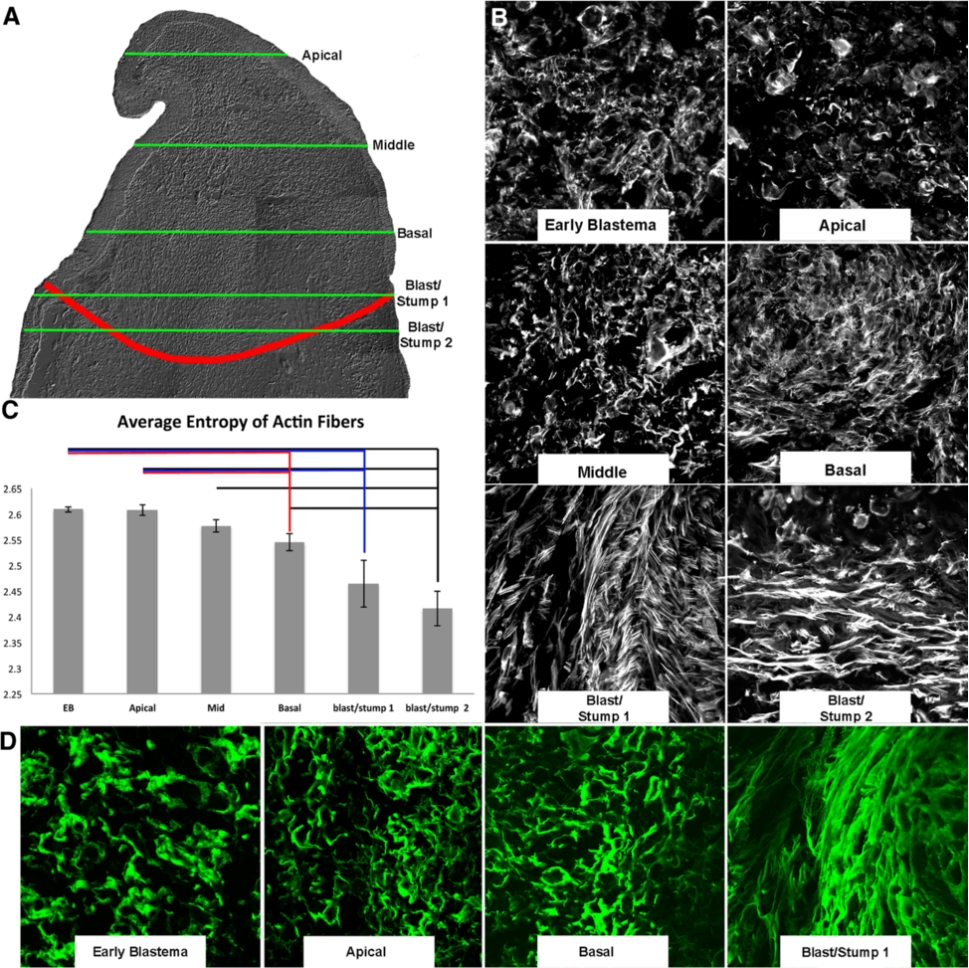
Characterization of the actin cytoskeleton and extracellular matrix (ECM) during regeneration. **a** A DIC image of a late bud blastema transverse section indicating where the sagittal sections in (**b**, **d**) are located along the proximal/distal axis. **b** Phalloidin-rhodamine staining of sagittal sections through an EB, or from the Apical to basal regions of the LB. **c** Quantification of the organization of the actin cytoskeleton in images in (**b**). Error bars are the SEM, and students *t*-test was used to determine statistically relevant changes in organization (*p* < 0.002). **d** Immunodetection of the ECM molecule tenascin (Green) in EB, or apical to basal regions of the LB

The recent identification of blastema populations with varying degrees of positional stability led us to better characterize these cells and identify differences in cell behavior. We looked at the organization of the actin-cytoskeleton within the cells of the blastema mesenchyme, and the extracellular matrix (ECM) surrounding these cells (Fig. 1). The actin cytoskeleton was analyzed on sagittally oriented tissue sections from EB and LB stage blastemas that had been stained with phalloidin-rhodamine, and the degree of order (alignment) or disorder of actin filaments was quantified using automated image processing that computed the discrete entropy of the actin fibers (Fig. 1b, c). We found that the actin fibers in the populations of cells that are positionally plastic (i.e., EB blastema and apical-LB blastema) were short in length and disorganized such that they did not have any apparent polarity (Fig. 1b, c). The extracellular matrix surrounding these tissues, visualized by immunoflourescence staining of the ECM molecule tenascin, also appeared disorganized (Fig. 1d). In contrast, the average entropy of actin fibers in the basal-LB was lower (Fig. 1b, c), and the extracellular matrix appeared to be more organized (Fig. 1d). We note that similar differences in the density and apparent organization of the reticular lamina, which is composed of multiple ECM molecules including collagens, was observed in the apical and basal regions of limb blastema by Neufeld and Aulthouse [15]. Thus, our observations on tenascin organization likely represent the blastema ECM as a whole. In summary, the amount of tissue organization was relatively low in EB and apical-LB compared to basal-LB populations.

### Characterization of cell proliferation among blastema cell populations

**Fig. 2:**
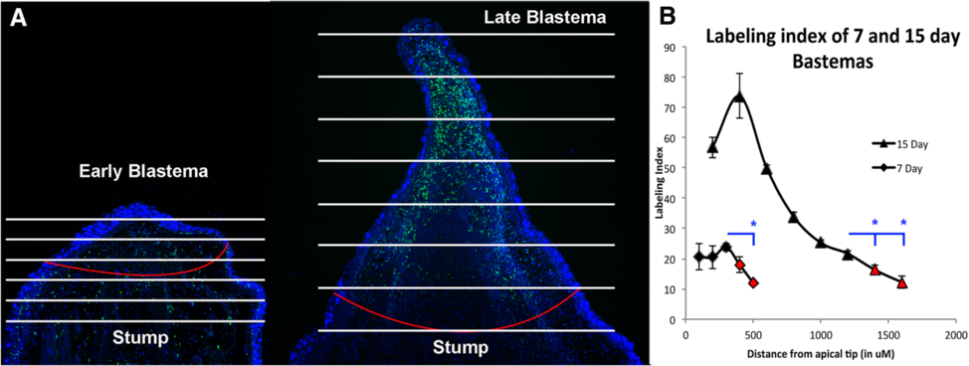
Characterization of cell proliferation in early and late blastemas. **a** Fluorescent images obtained on 10uM transverse sections of 7 day (early) and 15 day (late) blastemas that had been stained with DAPI (blue) for nuclei and Edu (green) for dividing cells. Animals were injected with Edu 3 h before tissue was harvested. White lines indicate the boundaries of each region that was quantified (100 uM segments for 7D samples, and 200 uM segments for 15D samples). **b** Histogram of the labeling index of digital sections from the apical (left) to basal (right) region of each type of blastema. The labeling index was quantified in 4 complete blastema sections for each sample. Error bars are the SEM, students *t*-test was used to determine statistically significant differences (*p* < 0.05) in the labeling index of the most basal blastema data-points and the stump data-points (red-filled symbols)

According to the Polar Coordinate model of regeneration, when cells with positional information from different locations in the limb are juxtaposed in a regeneration-competent environment, they induce a growth response that generates new cells with the missing positional information, a process known as intercalation [10, 11]. Thus, according to this model, cell proliferation is an integral part of the intercalary response during regeneration. To gain insight on when and where an intercalary response could be occurring in the developing blastema, and how this corresponds to the populations of cells with plastic or stabile positional information, we analyzed cell proliferation in EB blastemas and apical-LB blastemas. The proliferating cells in EB blastemas and LB blastemas were analyzed by calculating the percentage of cells in different regions of the blastema mesenchyme that incorporated EdU into newly synthesized DNA (Fig. 2). We found that the labeling index throughout the mesenchyme of the EB blastema was approximately 20 % (Fig. 2b). At the LB stage, the overall amount of cell proliferation was higher, and the labeling index varied greatly depending on whether the cells were located apically or basally. At the apical-most tip of the LB blastema, approximately 50 % of the cells were labeled. The labeling index increased to above 80 % in the cell population just proximal to these apical-most cells. The labeling index then gradually decreased in the basal region of the blastema (Fig. 2b). Similar results have been reported in regenerating amphibian limb blastemas and developing mouse limb buds [16, 17]. The labeling index in the stump tissue closest to both EB and LB blastemas was around 12 % (Fig. 2b); this is lower than the overall labeling index of the blastema mesenchyme, but higher than the labeling index that has been reported in uninjured limb tissues [18, 19].

Together, these results show that EB and apical-LB blastema populations have increased amounts of cell proliferation relative to the neighboring stump and basal-LB populations, respectively. It is possible that the increased amount of proliferation in EB and apical-LB tissue is associated with the process of intercalation. This idea is explored further in the discussion.

### Analysis of EB, apical-LB, and basal-LB blastema transcriptomes

**Fig. 3:**
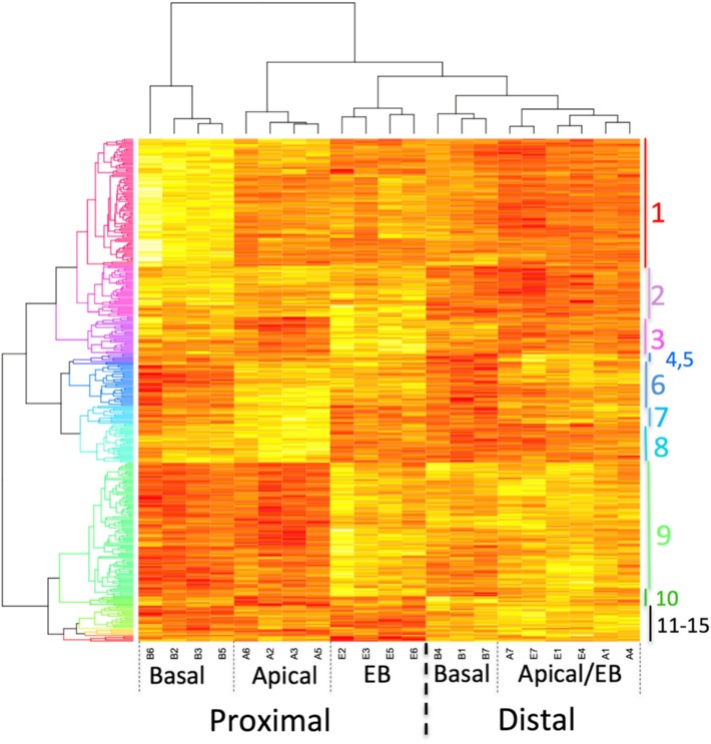
Significantly different gene expression in EB, apical-LB, and basal-LB mesenchymal tissues. The heatmap displays gene clusters (y-axis) and sample clusters (x-axis). Genes and samples were both clustered by average linkage clustering using 1-Pearson’s correlation as a distance measure. Only genes that tested statistically significant for the overall model were used for cluster analysis. Fifteen gene clusters were chosen and colored using R’s rainbow function [72]. Each colored box represents gene expression in a single sample for a single probe set with yellow indicating high expression and red indicating low expression. Samples grouped according to cell population, with the first major division occurring between proximal basal samples (samples B2, B3, B5, and B6) and all other samples. The next major division separates proximal apical samples from the remaining samples, and the following division separates proximal samples from the basal samples. Within distal samples, EB and apical-LB samples clustered together

To better understand mechanisms underlying the property of positional information in the regenerate, we tested for transcriptional differences among EB, apical-LB, and basal-LB blastema tissues that developed from proximal (mid-humerus) and distal (carpals) sites of limb amputation. We included proximal and distal blastema tissues in the experimental design because results from our previous study [9] predict that positionally plastic and positional stable cell populations should exhibit similar transcriptional patterns for genes associated with positional information, regardless of the site of amputation. Using a custom axolotl Affymetrix array (20,080 probesets) [20], 365 probesets were identified as significantly differently expressed using a two-way ANOVA model that treated blastema sample type (EB, apical-LB, basal-LB) and amputation site (proximal, distal) as fixed effects (Additional file 1: Table S1). To visualize these results in the form of a heatmap (Fig. 3), we performed two clustering analyses: 1) An objective methodology (see Methods and Additional file 2: Figure S1) was used to group the differently expressed probesets into 15 clusters; 2) Then, differently expressed probesets were used to cluster the blastema samples according to gene expression similarity. The interaction term (amputation site x blastema sample) was significant for 163 of the 365 probesets, indicating that gene expression varied among blastema populations as a function of amputation site. For example, 42 of the significant probesets in Cluster 1 were significant for the interaction term. Typically, Cluster 1 genes were expressed more highly in the proximal basal-LB sample than in all other samples, while expression estimates for proximal apical-LB and EB samples were similar to all three distal samples. As another example of gene x amputation site interaction, genes in Cluster 9 were similarly expressed in proximal basal-LB and apical-LB, but estimates tended to be higher and similar for proximal EB and all three distal samples. These results show that transcript abundances for many genes varied as both a function of apical-basal location within the blastema and proximal-distal location of the amputation site.

Only 24 probesets were significant for the amputation site main effect; the majority of these (*N* = 16) were assigned to Cluster 2. In comparison, 178 probesets were significant for the blastema sample main effect and these were distributed among all clusters except Cluster 12. As was observed for the list of significant interaction probesets, many of these genes were assigned to Clusters 1 and 9. The probesets from Cluster 1 (*N* = 49) registered transcript abundance estimates (within amputation sites) that were higher for basal-LB samples and lower for EB and apical samples. In contrast, probesets from Cluster 9 registered relatively higher expression estimates for EB and apical-LB samples than the basal-LB sample.

### Identifying a prioritized list of significant genes

Based on our previous study [9] and assuming that transcript abundances correlate with properties of positional information, we identified a high priority list of candidate genes (Additional file 3: Figure S3). We used t-tests (*p* < 0.05) to identify the set of probesets within the overall list of 365 that did not exhibit significantly different transcript abundances between proximal or distal EB and apical-LB tissues (*N* = 127). We then filtered this list to identify probesets that yielded statistically significant transcript abundances between EB and apical-LB compared to basal-LB tissues. To accomplish this filtering step, we used t-tests (*p* < 0.05) to identify significant probesets for the following four contrasts: Significantly different expression between EB and basal-LB blastemas arising from (1) distal and/or (2) proximal amputations; significantly different expression between apical-LB and basal-LB blastemas arising from (3) distal and/or (4) proximal amputations. A total of 67 significant probesets were identified for two or more of the contrasts (Additional file 4: Table S2). These results show that genes associated with developmental patterning (*bicd2*, *osr1*), chromatin structure (*jarid2*, *nfia*), cell proliferation, growth, and differentiation (*hbp1, cdkn2c, cndp2, gas2*), and major signaling pathways (FGF- *fgfrl1, fgfr3, fgfbp3*; RA- *crabp1*; BMP/TGFb- *tgfbr2, crim1*; WNT- *tcf7l2*) are expressed differently when comparing EB and apical-LB to basal-LB blastema.

### Annotation of significant genes

Of the 365 significant probesets, 325 had assigned gene names. We used these gene names to test for significantly enriched biological process ontology terms using Panther gene analysis tools [21] and a conservative familywise error rate (*p* < 0.0001). We ran separate analyses for each of the clusters in Fig. 3, however only Clusters 1 and 9, the two largest gene clusters, yielded significantly enriched terms. Eight Cluster 1 genes (*acan, sox6, fgfr3, osr1, gdf5, tgfbr2, lect1, myf5*), which were more highly expressed in basal-LB samples, significantly enriched the cartilage development ontology term (Bonferoni corrected prob = 0.008). These and other Cluster 1 genes are predicted to pattern tissues during early development, including structures associated with skeletal (e.g., *fgfrl1, foxc1, col9a2*) and nervous systems (*rgmb, rpe65, acan, fat3, sox6, fgfr3, gdf5, col9a2, tgfbr2, crim1, tcf7l2, pcdh15, gfra2, dact1, cdkn2c, gpr37L1, nrcam, ptprd, fabp7, lrig1, rgma, epha5, foxc1*) (Additional file 1: Table S1). A different set of related ontology terms were identified for Cluster 9 genes, which were more highly expressed in apical-LB and EB samples. Several ontogeny terms associated with extracellular matrix organization were enriched by an overlapping set of genes, including *serpinh1, mmp1, mmp2, mmp13, sparc, ctsk, fn1, itgad, nfkb2, ero1l* (Additional file 1: Table S1) (Bonferoni corrected prob = 0.004)*.* These genes encode proteins associated with matrix structure, disassembly, and collagen catabolism.

To further explore the significant gene list, we searched the literature using gene names as queries. We focused on genes involved in cell signaling and chromatin modification because both would seemingly be required to induce and maintain a plastic state. In Table 1, we highlight genes that fall within four general categories: cell signaling, chromatin modification, cell metabolism, and neural function/development. These genes encode proteins that function in FGF, ESRRG (estrogen-related receptor gamma), and mechanotransduction signaling pathways, as well as genes that modify histones via methylation, acetylation, and ubiquination.

**Table 1:** Genes with higher expression in basal-LB tissues

Potential role in blastema	Abbreviation	Gene name	Reference
Regulation of tissue differentiation	CAPSL	Calcyphosine-like	[77]
	CRABP1	Cellular retinoic acid binding protein 1	[78]
Skeletal differentation and development	ACAN	Aggrecan	[79]
	COL9A2	Collagen, type IX, alpha 2	[80]
	DACT1	Dishevelled-Binding Antagonist Of Beta-Catenin 1	[42]
	EMILIN3	Elastin Microfibril Interfacer 3	[52]
	FGFR3	Fibroblast growth factor receptor 3	[40]
	FOXC1	Forkhead Box C1	[81]
	GDF5	Growth differentiation factor 5	[82]
	ITGBL1	Integrin, beta-like 1 (with EGF-like)	[83]
	LECT1	Leukocyte cell derived chemotaxin 1	[84]
	TGFBR2	Transforming Growth Factor, Beta Receptor II	[85]
Muscle and tendon differentation	MYF5	Myogenic factor 5	[86]
	OLFM2	Neuronal Olfactomedin Related ER Localized Protein 2	[87]
	PLAC9	Placenta-specific 9	[88]
Axonal guidance and neural function	ACCN1	Acid-sensing ion channel 2	[89]
	CDH20	Cadherin 20, Type 2	[90]
	CPA6	Carboxypeptidase A6	[91]
	DAO	D-Amino-Acid Oxidase	[92]
	EPHA5	Ephrin type-A receptor 5	[93]
	ERMN	Ermin, ERM-Like Protein	[94]
	FAM19A5	Family With Sequence Similarity 19 (Chemokine (C-C Motif)-Like) Member 5	[95]
	FABP7	Fatty acid binding protein 7, brain	[96]
	GPR37L1	G Protein-Coupled Receptor 37 Like 1	[97]
	LPHN3	Latrophilin 3	[98]
	NRCAM	Neuronal cell adhesion molecule	[99]
	OTOS	Otospiralin	[100]
	PARK7	Parkinson protein 7	[101]
	RGMA	Repulsive Guidance Molecule Family Member A	[102]
	RGMB	RGM domain family, member B	[103]
	SLC7A10	Solute Carrier Family 7 (Neutral Amino Acid Transporter Light Chain, Asc System), Member 10	[104]
	TMEM47	Transmembrane Protein 47	[105]
Angiogenesis	ANGPTL1	Angiopoietin-related protein 1	[106]
	C1QTNF6	C1q and tumor necrosis factor related protein 6	[107]
	OSR1	Odd-skipped related 1	[108]
Regulation of tissue growth	CDKL1	Cyclin-dependent kinase-like 1	[109]
	CDKN2C	Cyclin-Dependent Kinase Inhibitor 2C	[110]
	LRIG3	Leucine-Rich Repeats And Immunoglobulin-Like Domains 3	[111]
	PDGFRL	Platelet-Derived Growth Factor Receptor-Like	[112]
	PTPRD	Protein tyrosine phosphatase, receptor type, D	[113]
	TMEM22	Solute carrier family 35 member G2	[114]
Genes with higher expression in EB and apical-LB tissues			
Cell Signaling	ESRRG	Estrogen-related receptor gamma	[115]
	FAM150A	Family With Sequence Similarity 150, Member A	[116]
	FGF8	Fibroblast Growth Factor 8	[117]
	G3BP2	Ras GTPase-activating protein-binding protein 2	[22]
DNA Repair and Modification	HABP4	Intracellular hyaluronan-binding protein 4	[118]
	JARID2	Jumonji, AT Rich Interactive Domain 2	[62]
	OBFC2A	NABP1 nucleic acid binding protein 1	[119]
	ZMYM2	Zinc Finger, MYM-Type 2	[120]
Ubiquination	ASB6	Ankyrin repeat and SOCS box protein 6	[121]
	SENP1	SUMO1/sentrin specific peptidase 1	[122]
	SPSB4	SplA/Ryanodine Receptor Domain And SOCS Box Containing 4	[123]
	UBA3	Ubiquitin-like modifier activating enzyme 3	[124]
Transcriptional Regulation and RNA processing	HBP1	HMG-Box Transcription Factor 1	[125]
	NR2C1	Nuclear receptor subfamily 2, group C, member 1	[126]
	SFRS2	Serine/Arginine-Rich Splicing Factor 2	[127]
	SNRPA1	Small Nuclear Ribonucleoprotein Polypeptide A	[128]
	ZNF674	Zinc Finger Protein 674	[129]
Translation	ETF1	Eukaryotic Translation Termination Factor	[130]
	ISG20L2	Interferon stimulated exonuclease gene 20 kDa-like 2	[131]
	PUS3	Pseudouridylate Synthase 3	[132]
Mitochondrial Function	C21orf2	Chromosome 21 Open Reading Frame 2	[133]
	DNM1L	Dynamin-1-like protein	[134]
	TBC1D20	TBC1 Domain Family, Member 20	[135]
	WARS2	Tryptophanyl TRNA Synthetase 2, Mitochondrial	[136]
	YARS2	Tyrosyl-tRNA synthetase 2, mitochondrial	[137]
Nerve Related	MAPK6	Mitogen-Activated Protein Kinase 6	[138]
	MLLT11	Myeloid/Lymphoid Or Mixed-Lineage Leukemia (Trithorax Homolog, Drosophila); Translocated To, 11	[139]
	RTN4	Reticulon 4	[140]
	SMN1	Survival of motor neuron 1, telomeric	[141]

## Discussion

### ECM organization as a mechanism for positional plasticity and stability

We observed that the gene *G3BP2* (Ras GTPase-activating protein-binding protein 2), which is part of a Twist1-G3BP2 mechanotransduction pathway, was expressed at higher levels in EB and apical-LB populations relative to basal-LB and stump populations. G3BP2 prevents Twist1 translocation to the nucleus, which results in activation of genes involved in differentiation [22]. Twist1 signaling is also regulated by matrix stiffness such that high stiffness in tissues results in G3BP2 release of Twist1, and activation of target genes. In the current study, we observed that the extracellular matrix molecule tenascin is more organized in the basal-LB tissue as compared to both EB and apical-LB tissue (Fig. 1); similar observations have been made for the blastema ECM as a whole [15]. It therefore is possible that the increased organization of the ECM in the basal-LB tissue alters Twist1-G3BP2 interactions, leading to an increase in Twist 1 nuclear translocation and expression of genes that promote differentiation in the blastema. Moreover, the increased abundance of genes involved in degrading the extracellular matrix (*mmp1, mmp2, mmp3, mmp13, ctsk*) that we have observed in EB and apical-LB could be upstream of G3BP2-mediated inhibition of differentiation in these plastic populations. Further experiments will be needed to test this hypothesis.

### The intercalary response and positional plasticity

According to the Polar Coordinate Model of regeneration, the process of intercalation drives tissue growth. In our study we discovered that the amount of cell proliferation was higher in the EB and apical-LB populations relative to basal-LB and stump populations. Thus, we hypothesize that the intercalary response is occurring in the plastic blastema populations (i.e., EB and apical-LB). If this is true, then positional plasticity could be a consequence of intercalation. As we have explained previously, a number of genes that regulate epigenetic modifications and chromatin structure are enriched in plastic blastema populations. Given that positional information is epigenetically encoded [13, 23, 24], it is likely that the establishment of the position-specific epigenome in the newly generated cells is mediated by some of these genes and has a temporal component.

Our findings provide candidate genes and pathways that maybe involved in the regulation of cell proliferation. Our transcriptional analysis reveals that *cndp2*, which promotes cell proliferation, is more highly expressed in EB and apical-LB blastema populations. EGFR signaling is required for CNDP2 mediated proliferative activity [23] and is activated during regeneration [25]. Additionally, EGFR activity is induced by matrix metalloprotease activity in the regenerate [26]. We observed increased expression of a number of metalloproteases (*mmp1, mmp2, mmp3, mmp13*) in EB and apical-LB populations. These metalloproteases could be upstream of a CNDP2-mediated growth response during intercalation. Likewise, we also discovered that two growth inhibitory factors, *cdkn2c* and *gas2* [27, 28], were more highly expressed in the basal-LB population. These results suggest the operation of mechanisms to inhibit growth in blastema tissues that are differentiating and not participating in a proliferation response.

### Candidate genes and pathways for positional plasticity and stability

Several transcriptional studies of limb regeneration have been performed in recent years [29–33]. In all studies to date, mRNAs were isolated from partial or entire blastemas, including in some cases underlying mature stump tissue. While we did not achieve the temporal resolution of the Voss et al. 2015 analysis, our study is the first to compare global patterns of transcription among spatial domains of a blastema. Our results show that transcript abundances vary as a function of apical/basal position within the blastema. We also compared transcription among different blastema cell populations that were collected after performing proximal and distal amputation. Almost half of the genes were significant for the interaction term in our statistical model, thus suggesting that gene expression varied among blastema populations as a function of amputation site. If the proteins encoded by these genes contribute to the property of positional plasticity that is shared between EB and apical-LB cell populations, then this property may not correlate well with transcript abundance. However, we caution that many of the interaction gene effects were identified as significant because of our unbalanced experimental design – more replicates were collected and analyzed for proximal amputations, thus more statistical power was available to detect significant differences. Consistent with this interpretation, 324 post-hoc t-tests performed were significant when contrasting proximal cell population samples, while only 64 t-tests were significant when contrasting distal cell population samples. This pattern was also observed for genes identified as significant for the cell population term in the statistical model – 322 t-tests performed among proximal cell population samples were found to be significant compared to only 116 t-tests for distal cell population samples. Finally, only 24 genes were identified as significant for the amputation site term in the statistical model. While it seems likely that some genes maybe expressed differently between distal and proximal blastema cell populations, including genes that associate with positional plasticity, further studies with more replicate samples and finer proximal-distal and temporal sampling will be needed to rigorously test this idea. At any rate, it will be important in future transcriptional studies of limb regeneration to more finely investigate temporal and spatial components of variation.

While we consider all of 365 significant probesets identified from our statistical tests as potentially providing perspective about the molecular basis of positional information, we prioritized these genes further using t-tests. Working under the assumption that transcript abundance correlates with properties of positional information, we identified a high priority list of 65 genes. Highlighting genes primarily from this list, we discuss candidate genes and pathways that may underlie the properties of positional stability and plasticity.

Memory of positional information is a property that appears to be exclusive to fibroblasts in connective tissues throughout the body. This is based on transcriptional and epigenetic data [13, 23, 34], and the ability of connective tissues to induce the formation of new limb structures [10–12]. The EB-blastema mesenchyme is composed largely of cells of fibroblast origin [35] and thus the expression profiles in the EB mesenchyme should predominately reflect gene expression in blastema cells of fibroblast origin in a positionally plastic state. On the other hand, determining the transcriptional profiles that are linked with fibroblast-derived cells that have stabile positional information is more complicated because the basal-LB tissue is more heterogeneous, being composed of cells of multiple tissue origins [35–37]. However, since fibroblast-derived blastema cells contribute to the skeletal elements in the regenerate [36], the expression of genes involved in the development of cartilage/bone is likely associated with fibroblast-derived cells as they differentiate into these tissues. With this in mind, we discuss how genes that were significantly enriched in EB and apical-LB compared to basal-LB populations could control/affect the stability of positional information in fibroblast-derived blastema cells.

#### FGF signaling

FGF pathway members showed quantitative variation between positionally plastic and stable populations. At the EB stage, *fgf8* expression initiates in basal cells of the wound epidermis and underlying blastema mesenchyme cells [38]. While *fgf8* did not make the prioritized list of 65, expression was significantly higher in EB and apical-LB samples than basal-LB samples. This same pattern was observed for *ebna1bp2,* which encodes an FGF binding protein [39]*.* Inverse to these patterns, we observed higher expression of two FGF receptors (*fgfr3, fgfrl1*) and a binding protein that regulates FGF receptor signaling (*fgfbp3)* in the basal compartment of the LB blastema. These patterns suggest that FGF pathway components vary quantitatively between apical and basal compartments of a blastema. We hypothesize that these quantitative differences may contribute to a gradient mechanism that maintains positional plasticity in EB and apical-LB compartments, but promotes cellular differentiation and positional stability in the basal-LB compartment.

In support of this hypothesis, *fgfr3* is expressed exclusively in differentiating chondrocytes in the proximal region of the developing mouse limb bud [40, 41]. One of the targets of FGF signaling during limb development is *dact1,* which we found to be highly expressed in basal-LB tissue. *Dact1* is expressed in the limb bud mesenchyme, specifically in the developing cartilage [42–44]. Knockout of *dact1* in mouse disrupts Planar Cell Polarity signaling [45], which plays a role in controlling the size and shape of the limb during limb bud morphogenesis [46]. FGFR3 and DACT1 colocalize to the same cells in developing limb bud, and FGF signaling activates the expression of *dact1* [47]. SOX9, which is upstream of FGFR3 and COL9A2 expression and binds to cartilage-specific enhancers that regulate *acan* and *col2A* transcription [48], was also expressed more highly in basal-LB tissue. We observed an increase in the expression of both *acan* and *col2A* in the basal compartment, supporting the idea that similar mechanisms are underway in basal-LB blastema cells. It is possible that Sox9 activates the expression of *fgfr3* and *dact1* to induce the differentiation blastema cells into chondrocytes, and thus could also be linked to the stabilization of positional information in those cells during limb regeneration.

#### TGFb/BMP signaling

Given the role of TGFb/BMP signaling in the differentiation and development of the skeletal elements, it is not surprising to see an increased expression of a number of genes involved in this pathway in the basal compartment (*tgfbr1, emilin3 crim1, gdf5 and osr1)* where cartilage differentiation is actively underway. *Tgfbr1*, encodes for a TGFb receptor that plays an important role in skeletal development and regeneration in mammals and amphibians [49–51]. Emilin3 acts as a TGFb antagonist, and is expressed in the periskeletal tissue in developing limb buds [52]. *Crim1* encodes for a gene that acts as a BMP antagonist by negatively regulating the processing and secretion of BMPs [53]. However, Crim1 is expressed in multiple tissues throughout the developing limb [54] and thus it is difficult to understand how this gene functions to regulate BMP signaling. Conversely, Gdf5 (aka BMP14) is a BMP-ligand that is expressed in, and is required for, developing and regenerating limb joints [55, 56]. Osr1 expression in the limb bud mesenchyme is essential for synovial joint formation, in part by maintaining the expression of *gdf5* in the forming joint [57]. Interestingly, FGF signaling is required for *osr1* expression in Xenopus embryos [58], and it is possible that some of the aforementioned FGF-related genes could play a role in the induction of *osr1* expression in blastema cells as they differentiate into cartilage.

On the other hand, the inhibition of TGFb/BMP signaling could be required to prevent differentiation of positionally plastic cells. We observed higher expression of *esrrg* in the EB and apical-LB samples as compared to the basal-LB sample. This gene encodes an orphan nuclear receptor that binds to both the estrogen response element and the steroidogenic factor 1 response element, and activates gene expression in the absence of ligand [59]. ESRRG negatively regulates BMP-2 induced bone differentiation and formation in mammals [60], and thus this gene would be expected to function in preventing BMP-2 mediated differentiation of blastema cells.

#### Chromatin structure and epigenetic regulation

Large-scale chromatin rearrangements occur in cells of the uninjured limb as they dedifferentiate and become blastema cells, such that the chromatin becomes less densely packed and more euchromatic [61]. Consistent with this observation, genes that regulate epigenetic modifications (*jarid2)* and chromatin structure (*zym2, habp4)* are more highly expressed in EB and apical-LB tissues. Of note, JARID2 regulates differentiation of embryonic stem (ES) cells and cells within developing embryos by associating with PRC2 and inhibiting the trimethylation of histone H3 (H3K27Me3) [62]. JARID2 also plays a role in the recruitment of PRC2 to *Hox* loci in differentiating embryonic stem cells [63]. Presumably JARID2 plays a similar role in the blastema and is involved in the re-programming of positional information in the plastic blastema population. *NFIA*, which encodes for a transcription factor that can interact with and modify nucleosomal structure [64, 65], is enriched in the basal-LB tissue. This is interesting because NFIA promotes the differentiation of multiple tissue types [66–68], and likely has a similar function in the progenitor cells within the basal compartment. Altogether these observations indicate that genes that are involved with regulating chromatin structure and the epigenome are active in both plastic and stabilized blastema populations.

#### Ubiquination

*Spsb4* and *asb6* encode for proteins that are involved in protein ubiquination, and are more highly expressed in positionally plastic EB and apical-LB blastema populations. Since ubiquination is an important part of protein turnover [69], it is possible that the expression of these genes is linked to the increased metabolism of these dynamic cell populations (Fig. 2 and [70]). In addition, histone ubiquination is an important modification that alters nucleosome, and therefore chromatin structure [71]. Thus these ubiquitin–related genes may play a role in re-programming blastema cells as well as in cellular housekeeping.

#### Neural development and function

Above, we focused on genes that are likely to be expressed in fibroblast-derived blastema cells because they retain positional information. However, we note that a number of genes associated with neural development and function were expressed more highly in the basal-LB sample. These genes include *asic2, cdh20, fabp7, fam19a5, llphn2, megf10, pcdh8, rgma, rgmb, scfd2, serpine2, tmem47.* It is unclear whether these expression patterns reflect increased expression in the basal compartment, or a decreased expression the apical-LB compartment. However, if the abundance of these transcripts is indicative of neural activity in these tissues, these activities appear to be higher in basal-LB than apical-LB mesenchyme. We speculate that this could be a result of the formation of new neural connections with the differentiating tissues in this region.

## Conclusion

In a previous study we discovered that positional information is unstable (or plastic) in EB and apical-LB populations, and is stabilized in basal-LB tissue [9]. To identify candidate mechanisms that regulate the stability of positional information in the blastema we compared EB and apical–LB samples to the basal-LB sample. We observed that genes associated with negative and positive regulation of cell differentiation were more abundantly expressed in plastic and stabilized populations, respectively. This suggests that the stability of positional information in blastema cells maybe associated with the regulation of signaling pathways for cellular differentiation. Our results also suggest that positional stability is a property of cells with increased ECM organization and associated gene expression. This observation likely reflects the increased amount of differentiating cells in the basal compartment, although it is also possible that ECM-linked mechanisms could regulate differentiation. From the current study it is unclear whether processes of cell differentiation and the stabilization of positional information are linked genetically, or whether the same molecular mechanism is utilized to “hardwire” both cellular and positional identity. We speculate that the latter possibility is likely, given the role of epigenetic modifications in the specification of both of these properties. Lastly, plastic blastema populations are associated with increased cell proliferation and higher expression of genes that promote growth. Since the theory of intercalation is based on the idea that positional discontinuity drives tissue growth, we posit that increased proliferation is an intercalary outcome. Altogether, our results provide an important first-step toward better characterizing spatiotemporal changes that occur in the developing blastema, and identifying candidate mechanisms that regulate the cellular property of positional-stability in the blastema during regeneration.

## Methods

### Animal husbandry

This study was carried out in accordance with the recommendations in the Guide for the Care and Use of Laboratory Animals of the National Institutes of Health. The experimental work was approved by the Institutional Animal Care and Use Committee of the University of California Irvine.

All of the experiments in this study were performed on the Mexican axolotl (*Ambystoma mexicanum*) measuring approximately 15–20 cm from snout to tail tip (Ambystoma Genetic Stock Center, UKY). Animals were anesthetized using a 0.1 % solution of MS222 (Ethyl 3-aminobenzoate methanesulfonate salt, Sigma), pH 7.0. To initiate regeneration, animals were either amputated just proximal to the carpals (distal amputation), or at the proximal end of the humerus (proximal amputation).

### Tissue collection

The forelimbs and hindlimbs of animals were amputated at either the carpal level (distal), or at the upper arm (proximal). When the blastemas had reached early bud stage, the EB blastema mesenchymal tissue was harvested by gently teasing away the wound epithelium, and conservatively collecting blastema tissue, being careful not to collect mature limb tissue. On the same animals, the blastemas were allowed to regenerate until they reached LB stage. Similar to EB collection, the wound epithelium was carefully removed from LB blastemas. Blood vessels are sparse in the apical region of the LB, and the lack of blood vessels was used as an ocular cue for apical tissue collection. Basal-LB tissue was then collected conservatively as to not include stump tissue. See [9] for a more detailed description of how EB, apical-LB, and basal-LB tissue are collected. Stump tissue was collected from the same proximal/distal locations as the blastema tissues on unamputated animals, and included deep and superficial tissues in the limb. For each sample, tissues were pooled from 8 blastemas. Microarray was performed by the University of Kentucky microarray core using 3–4 independent biological replicate samples per each tissue type.

### Microarray analysis

Samples were processed by RMA using Affymetrix Expression Console (Affymetrix, Santa Clara, CA). Samples were divided into three groups: early blastema (E) apical late blastema (A), and basal late blastema (B). All probesets that were consistently expressed below the bottom quartile of mean expression across all samples were removed from the analysis. Samples were also separated based on whether they were gathered from a distal (D) or proximal (P) amputation. Each probeset was fit to the generalized linear model of y = Β_o_c + Β_1_a + Β_2_ c * a + i + ε), where y was the measured log2 transformed intensity from the microarray, Β_0_, Β_1_, and Β_2_.were coefficients for the blastema cell type (c), amputation site (a), and blastema cell type by amputation site interaction effects, i was the intercept term, and ε was the normally distributed error. R was used to extract p-values from the full model using the lm function, as well as perform a two-way analysis of variance using the aov function to derive p-values associated with each main effect and the interaction term. Post-hoc t-tests to compare cell populations within an amputation site were also performed in R. The hclust and heatmap functions were used to cluster both samples and probesets using average linkage clustering on Pearson’s correlation [72]. Changes in gene expression were considered significant if the full model passed an FDR correction at 0.05, and post hoc t-tests were evaluated at *p* < 0.05. Probesets that were identified as showing a statistically significant change were clustered by average linkage clustering using 1-Pearson's correlation as a distance metric. Join distances were plotted as a function of the number of clusters to identify at what stage dissimilar expression profiles would be joined into the same group. From this plot, 15 clusters were selected and used [73, 74].

Gene enrichment analysis was performed using PANTHER gene expression tools [21] and the complete set of Biological Process gene ontology terms. The reference file for analysis was the complete list of annotated probesets (i.e., probesets with gene name acronyms) from the axolotl Affymetrix microarray. A familywise error rate (*p* < 0.00001) was used to correct for multiple tests.

### Phalloidin and tenascin staining

Tissues for phalloidin-rhodamine and tenascin immunoflourescence were fixed, prepared for embedding in OCT, and cryosectioned into 10um slices. Sections were washed, and permeabilized using 0.01 % Triton-X prior to staining procedure. To stain the actin cytoskeleton, phalloidin-rhodamine (Cytoskeleton Inc., Denver, Co.) (14 uM) diluted 1:100 in PBS was incubated on sections for 1 h in the dark prior to washing and stabilizing with Vectashield mounting medium with DAPI (Vector Laboratories, Burlingham, Ca). Tenascin immunofluorescence was performed using the MT1 anti-newt-tenascin monoclonal antibody. The MT1 antibody, developed by Roy A. Tassava was obtained from the Developmental Studies Hybridoma Bank developed under the auspices of the NICHD and maintained by the University of Iowa, Department of Biology, Iowa City, IA 52242. Immunofluorescence was performed similar to [75] by incubating sections at 4 °C overnight with a 1:10 dilution in PBS of MT1 hybridoma media, washing, and a 1 h incubation with goat-anti-mouse antibody conjugated to Alexa-488 (Invitrogen). Images were obtained using a 20x objective on a Zeiss LSM780 (2-photon) confocal microscope.

### Quantification of actin fiber characteristics

Confocal images were obtained of complete 10um deep, sagitally oriented, tissue sections of the blastema at different apical/basal positions, which were fluorescently stained for F-actin with phalloidin-rhodamine as described above. The tissue sections analyzed (Fig. 1b-c) were located approximately where the green lines indicate on Fig. 1a. The actin fiber orientations within each sagittal section were quantified using automated image processing implemented with a custom MATLAB script. The dominant orientation was estimated at each image pixel location by computing the image gradient using a Gaussian derivative filter (sigma = 2). The resulting orientation field was smoothed via standard methods [76] with a Gaussian filter (sigma = 10). To assess the differences in orientation across the spatial extent of each tissue section, the image was divided into small non-overlapping tiles (512x512 pixels) each covering approximately 150 square microns of tissue, and all together covered all of the mesenchymal tissue from each section. The distribution of gradient orientations in each tile was estimated using a histogram with 15 equal bins over the range of orientations (0–180°). Pixels with a gradient magnitude of less that 1 % of the maximum image intensity were excluded from analysis. This threshold served to eliminate locations in the image where no stained actin fibers were visible by eye (and where the corresponding orientation estimate is unstable). The discrete entropy of each histogram was computed as a summary statistic to measure the degree of order (alignment) or disorder of actin filaments within the region of tissue spanned by the tile.

The average of the discrete entropy was calculated for each apical/basal region of the blastema by calculating the average entropy of all of the tiles that were of only mesenchymal tissue (i.e., did not include epithelium) from a tissue section at each location. Figure 1c is a representative data set of the quantification of the entropy values from individual tissue sections at different positions within a single blastema. The quantification of the actin cytoskeleton was performed on multiple blastemas that were sectioned either sagitally or transversally, and each showed the same trend of decreasing entropy in the increasingly basal tissues. The data from multiple blastemas was not combined because we could not ensure that the apical/basal location of each section was equivalent from blastema to blastema. In Additional file 5: Figure S2 we provide an example of the workflow on the analysis that was performed on a transverse tissue section of a late blastema. Note that the same apical/basal trend of actin organization was observed.

### Edu injection, detection, and quantification of labeling index

100 ug of Edu (Invitrogen, Carlsbad, Ca) was injected intraperitoneal in the flank of the animal 3 h prior to tissue collection. Tissues were fixed in 3.7 % PFA solution, embedded in paraffin, and detected using the manufactures protocol. Vectashield with DAPI (Vector Laboratories, Burlingham, Ca) was used to stain all nuclei. A tile scan of the entire blastema was obtained using a 20x objective on a Zeiss LSM780 (2-photon) confocal microscope. To quantify the % labeling index of the blastema mesenchyme, the wound epithelium was removed digitally using Photoshop, and the blastema was divided into digital sections approximately 200 uM along the proximal/distal axis. Edu and DAPI stained nuclei were manually counted using the cell counter in the ‘analyze’ plug-in on ImageJ. The % labeling index was determined for each digital section by calculating the ratio of Edu positive cells to total number of cells, and multiplying by 100.

## Availability of supporting data

The microarray data files supporting the results of this article are available for download at Sal-Site http://www.ambystoma.org/genome-resources/20-gene-expression.

## Additional files

Additional file 1: Table S1. Probesets with interaction term *p* < 0.05. (XLSX 227 kb) Additional file 2: Figure S1. Selection of 15 clusters of gene expression in EB, apical-LB, and basal-LB in different locations on the P/D limb axis. Join distance (1-Pearson’s correlation, y-axis) plotted as a function of number of clusters. After fifteen clusters, join distances reach an asymptote, suggesting that using more than fifteen groups provides little additional information (small distance indicates separating similarly expressed genes), while using more than fifteen clusters results in joining dissimilar groups (high distance). (TIFF 1203 kb) Additional file 3: Figure S3. Diagram showing the statistical strategy used to identify 67 candidate probesets that were expressed differently between EB/apical-LB and basal-LB. We took the 365 significant genes identified using two-way ANOVA and filtered them using t-tests (α = 0.05). First, we identified all probesets (*N* = 127) that did not show a significant amputation site effect (*p* > 0.05) between proximal and distal EB samples, or between proximal and distal basal-LB samples. We then identified all probesets (*N* = 67) that were significant (*p* < 0.05) when separately comparing EB and apical–LB samples to the basal-LB sample. (TIFF 395 kb) Additional file 4: Table S2. Prioritized list of 67 probesets that registered signficant differences in expression between EB/Apical-LB versus Basal-LB. (XLSX 55 kb) Additional file 5: Figure S2. Workflow of actin quantification on a transverse section of a LB-stage blastema. (A) Confocal image of a transverse section of a LB blastema that had been stained with phalloidin-rhodamin for F-actin (Red). (B) The actin fiber orientations within the blastema mesenchyme of the tissue section was quantified using automated image processing (described in Methods section). Each color represents the orientation of fibers toward a specific direction. For example, red colored fibers are oriented along the proximal/distal axis. (C) The image was divided into small non-overlapping tiles (512 × 512 pixels) each covering approximately 150 square microns of tissue, and all together covered all of the mesenchymal tissue from each section. (D) The discrete entropy of each histogram was computed as a summary statistic to measure the degree of order (alignment) or disorder of actin filaments within the region of tissue spanned by the tile. (E) Histogram representing the average entropy of the tiles from each region of the blastema (Blast/stump, Basal, Mid, Apical, as indicated in (D)). The error bars are the SEM, and Student’s *t*-test was used to determine statistically signficant changes in organization (*p* < 0.01). (TIFF 538 kb)

## Abbreviations

EB: early-bud blastema
LB: late-bud blastema
ECM: extracellular matrix
RA: retinoic acid
Tbx: T-box
EdU: 5-ethynyl-2′-deoxyuridine, gene IDs are located in supplemental tables

## References

[1] Stocum DL (1980). Autonomous development of reciprocally exchanged regeneration blastemas of normal forelimbs and symmetrical hindlimbs. J Exp Zool.

[2] Gardiner DM, Blumberg B, Komine Y, Bryant SV (1995). Regulation of HoxA expression in developing and regenerating axolotl limbs. Development.

[3] Roensch K, Tazaki A, Chara O, Tanaka EM (2013). Progressive specification rather than intercalation of segments during limb regeneration. Science.

[4] Niazi IA, Pescitelli MJ, Stocum DL (1985). Stage-dependent effects of retinoic acid on regenerating urodele limbs. W Roux' Archiv f Entwicklungsmechanik.

[5] McCusker C, Lehrberg J, Gardiner D (2014). Position-specific induction of ectopic limbs in non-regenerating blastemas on axolotl forelimbs. Regeneration.

[6] Maden M (1983). The effect of vitamin A on the regenerating axolotl limb. J Embryol Exp Morph.

[7] Ragsdale CW, Gates PB, Hill DS, Brockes JP (1993). Delta retinoic acid receptor isoform δ1 is distinguished by its exceptional N-terminal sequence and abundance in the limb regeneration blastema. Mech Dev.

[8] Monaghan JR, Maden M (2012). Visualization, of retinoic acid signaling in transgenic axolotls during limb development and regeneration. Dev Biol.

[9] McCusker CD, Gardiner DM (2013). Positional information is reprogrammed in blastema cells of the regenerating limb of the axolotl (Ambystoma mexicanum). PLoS ONE.

[10] French V, Bryant PJ, Bryant SV (1976). Pattern regulation in epimorphic fields. Science.

[11] Bryant SV, French V, Bryant PJ (1981). Distal regeneration and symmetry. Science.

[12] Endo T, Bryant SV, Gardiner DM (2004). A stepwise model system for limb regeneration. Dev Biol.

[13] Rinn JL, Wang JK, Allen N, Brugmann SA, Mikels AJ, Liu H, Ridky TW, Stadler HS, Nusse R, Helms JA, Chang HY (2008). A dermal HOX transcriptional program regulates site-specific epidermal fate. Genes Dev.

[14] McCusker CD, Gardiner DM (2014). Understanding positional cues in salamander limb regeneration: implications for optimizing cell-based regenerative therapies. Dis Model Mech.

[15] Neufeld DA, Aulthouse AL (1986). Association of mesenchyme with attenuated basement membranes during morphogenetic stages of newt limb regeneration. Am J Anatomy.

[16] Smith AR, Crawley AM (1977). The pattern of cell division during growth of the blastema of regenerating newt forelimbs. J Embryol Exp Morphol.

[17] Boehm B, Westerberg H, Lesnicar-Pucko G, Raja S, Rautschka M, Cotterell J, Swoger J, Sharpe J (2010). The role of spatially controlled cell proliferation in limb bud morphogenesis. PLoS Biol.

[18] Wallace H, Maden M (1976). The cell cycle during amphibian limb regeneration. J Cell Sci.

[19] Lehrberg J, Gardiner DM (2015). Regulation of Axolotl (Ambystoma mexicanum) limb blastema cell proliferation by nerves and BMP2 in organotypic slice culture. PLoS ONE.

[20] Huggins P, Johnson CK, Schoergendorfer A, Putta S, Bathke AC, Stromberg AJ, Voss SR (2012). Identification of differentially expressed thyroid hormone responsive genes from the brain of the Mexican Axolotl (Ambystoma mexicanum). Comp Biochem Physiol C Toxicol Pharmacol.

[21] Mi H, Muruganujan A, Thomas PD (2013). PANTHER in 2013: modeling the evolution of gene function, and other gene attributes, in the context of phylogenetic trees. Nucleic Acids Res.

[22] Wei SC, Fattet L, Tsai JH, Guo Y, Pai VH, Majeski HE, Chen AC, Sah RL, Taylor SS, Engler AJ, Yang J (2015). Matrix stiffness drives epithelial-mesenchymal transition and tumour metastasis through a TWIST1-G3BP2 mechanotransduction pathway. Nat Cell Biol.

[23] Rinn JL, Kertesz M, Wang JK, Squazzo SL, Xu X, Brugmann SA, Goodnough LH, Helms JA, Farnham PJ, Segal E, Chang HY (2007). Functional demarcation of active and silent chromatin domains in human HOX loci by noncoding RNAs. Cell.

[24] Chang HY, Chi J-T, Dudoit S, Bondre C, van de Rijn M, Botstein D, Brown PO (2002). Diversity, topographic differentiation, and positional memory in human fibroblasts. Proc Natl Acad Sci U S A.

[25] Xue C, Zhang Z, Yu H, Yu M, Yuan K, Yang T, Miao M, Shi H (2014). Up-regulation of CNDP2 facilitates the proliferation of colon cancer. BMC Gastroenterol.

[26] Blassberg RA, Garza-Garcia A, Janmohamed A, Gates PB, Brockes JP (2011). Functional convergence of signalling by GPI-anchored and anchorless forms of a salamander protein implicated in limb regeneration. J Cell Sci.

[27] Hosokawa H, Tanaka T, Kato M, Shinoda K, Tohyama H, Hanazawa A, Tamaki Y, Hirahara K, Yagi R, Sakikawa I, Morita A, Nagira M, Poyurovsky MV, Suzuki Y, Motohashi S, Nakayama T (2013). Gata3/Ruvbl2 complex regulates T helper 2 cell proliferation via repression of Cdkn2c expression. Proc Natl Acad Sci.

[28] Zhang T, Dayanandan B, Rouiller I, Lawrence EJ, Mandato CA (2011). Growth-arrest-specific protein 2 inhibits cell division in Xenopus embryos. PLoS ONE.

[29] Monaghan JR, Epp LG, Putta S, Page RB, Walker JA, Beachy CK, Zhu W, Pao GM, Verma IM, Hunter T, Bryant SV, Gardiner DM, Harkins TT, Voss SR (2009). Microarray and cDNA sequence analysis of transcription during nerve-dependent limb regeneration. BMC Biol.

[30] Stewart R, Rascón CA, Tian S, Nie J, Barry C, Chu L-F, Ardalani H, Wagner RJ, Probasco MD, Bolin JM, Leng N, Sengupta S, Volkmer M, Habermann B, Tanaka EM, Thomson JA, Dewey CN (2013). Comparative RNA-seq analysis in the unsequenced axolotl: the oncogene burst highlights early gene expression in the blastema. PLoS Comput Biol.

[31] Monaghan JR, Athippozhy A, Seifert AW, Putta S, Stromberg AJ, Maden M, Gardiner DM, Voss SR (2012). Gene expression patterns specific to the regenerating limb of the Mexican axolotl. Biology Open.

[32] Knapp D, Schulz H, Rascón CA, Volkmer M, Scholz J, Nacu E, Le M, Novozhilov S, Tazaki A, Protze S, Jacob T, Hubner N, Habermann B, Tanaka EM (2013). Comparative transcriptional profiling of the axolotl limb identifies a tripartite regeneration-specific gene program. PLoS ONE.

[33] Voss SR, Palumbo A, Nagarajan R, Gardiner DM, Muneoka K, Stromberg AJ, Athippozhy AT (2015). Gene expression during the first 28 days of axolotl limb regeneration I: Experimental design and global analysis of gene expression. Regeneration.

[34] Rinn JL, Bondre C, Gladstone HB, Brown PO, Chang HY (2006). Anatomic Demarcation by Positional Variation in Fibroblast Gene Expression Programs. PLoS Genet.

[35] Muneoka K, Fox WF, Bryant SV (1986). Cellular contribution from dermis and cartilage to the regenerating limb blastema in axolotls. Dev Biol.

[36] Kragl M, Knapp D, Nacu E, Khattak S, Maden M, Epperlein HH, Tanaka EM (2009). Cells keep a memory of their tissue origin during axolotl limb regeneration. Nature.

[37] Nacu E, Glausch M, Le HQ, Damanik FFR, Schuez M, Knapp D, Khattak S, Richter T, Tanaka EM (2013). Connective tissue cells, but not muscle cells, are involved in establishing the proximo-distal outcome of limb regeneration in the axolotl. Development.

[38] Han MJ, An JY, Kim WS (2001). Expression patterns of Fgf-8 during development and limb regeneration of the axolotl. Dev Dyn.

[39] Reimers K, Antoine M, Zapatka M, Blecken V, Dickson C, Kiefer P (2001). NoBP, a nuclear fibroblast growth factor 3 binding protein, is cell cycle regulated and promotes cell growth. Mol Cell Biol.

[40] Davidson D, Blanc A, Filion D, Wang H, Plut P, Pfeffer G, Buschmann MD, Henderson JE (2005). Fibroblast Growth Factor (FGF) 18 Signals through FGF Receptor 3 to Promote Chondrogenesis. J Biol Chem.

[41] Wilkie AOM, Patey SJ, Kan S-H, van den Ouweland AMW, Hamel BCJ (2002). FGFs, their receptors, and human limb malformations: Clinical and molecular correlations. Am J Med Genet.

[42] Hammond KL, Hanson IM, Brown AG, Lettice LA, Hill RE (1998). Mammalian and Drosophila dachshund genes are related to the Ski proto-oncogene and are expressed in eye and limb. Mech Dev.

[43] Caubit X, Thangarajah R, Theil T, Wirth J, Nothwang HG, Rüther U, Krauss S (1999). Mouse Dac, a novel nuclear factor with homology to Drosophila dachshund shows a dynamic expression in the neural crest, the eye, the neocortex, and the limb bud. Dev Dyn.

[44] Ayres JA, Shum L, Akarsu AN, Dashner R, Takahashi K, Ikura T, Slavkin HC, Nuckolls GH (2001). DACH: genomic characterization, evaluation as a candidate for postaxial polydactyly type A2, and developmental expression pattern of the mouse homologue. Genomics.

[45] Mao Y, Mulvaney J, Zakaria S, Yu T, Morgan KM, Allen S, Basson MA, Francis-West P, Irvine KD (2011). Characterization of a Dchs1 mutant mouse reveals requirements for Dchs1-Fat4 signaling during mammalian development. Development.

[46] Wang B, Sinha T, Jiao K, Serra R, Wang J (2011). Disruption of PCP signaling causes limb morphogenesis and skeletal defects and may underlie Robinow syndrome and brachydactyly type B. Hum Mol Genet.

[47] Horner A, Shum L, Ayres JA, Nonaka K, Nuckolls GH (2002). Fibroblast growth factor signaling regulates Dach1 expression during skeletal development. Dev Dyn.

[48] Lefebvre V, Li P, de Crombrugghe B (1998). A new long form of Sox5 (L-Sox5), Sox6 and Sox9 are coexpressed in chondrogenesis and cooperatively activate the type II collagen gene. EMBO J.

[49] Loeys BL, Chen J, Neptune ER, Judge DP, Podowski M, Holm T, Meyers J, Leitch CC, Katsanis N, Sharifi N, Xu FL, Myers LA, Spevak PJ, Cameron DE, Backer JD, Hellemans J, Chen Y, Davis EC, Webb CL, Kress W, Coucke P, Rifkin DB, De Paepe AM, Dietz HC (2005). A syndrome of altered cardiovascular, craniofacial, neurocognitive and skeletal development caused by mutations in TGFBR1 or TGFBR2. Nat Genet.

[50] Liu J, Johnson K, Li J, Piamonte V, Steffy BM, Hsieh MH, Ng N, Zhang J, Walker JR, Ding S, Muneoka K, Wu X, Glynne R, Schultz PG (2011). Regenerative phenotype in mice with a point mutation in transforming growth factor beta type I receptor (TGFBR1). Proc Natl Acad Sci U S A.

[51] Levesque M, Gatien S, Finnson K, Desmeules S, Villiard E, Pilote M, Philip A, Roy S (2007). Transforming growth factor: beta signaling is essential for limb regeneration in axolotls. PLoS ONE.

[52] Schiavinato A, Becker A-KA, Zanetti M, Corallo D, Milanetto M, Bizzotto D, Bressan G, Guljelmovic M, Paulsson M, Wagener R, Braghetta P, Bonaldo P (2012). EMILIN-3, peculiar member of elastin microfibril interface-located protein (EMILIN) family, has distinct expression pattern, forms oligomeric assemblies, and serves as transforming growth factor β (TGF-β) antagonist. J Biol Chem.

[53] Wilkinson L, Kolle G, Wen DY, Piper M, Scott J, Little M (2003). CRIM1 regulates the rate of processing and delivery of bone morphogenetic proteins to the cell surface. J Biol Chem.

[54] Pennisi DJ, Wilkinson L, Kolle G, Sohaskey ML, Gillinder K, Piper MJ, McAvoy JW, Lovicu FJ, Little MH (2007). Crim1KST264/KST264 mice display a disruption of the Crim1 gene resulting in perinatal lethality with defects in multiple organ systems. Dev Dyn.

[55] Lee J, Gardiner DM (2012). Regeneration of Limb Joints in the Axolotl (Ambystoma mexicanum). PLoS ONE.

[56] Settle SH, Rountree RB, Sinha A, Thacker A, Higgins K, Kingsley DM (2003). Multiple joint and skeletal patterning defects caused by single and double mutations in the mouse Gdf6 and Gdf5 genes. Dev Biol.

[57] Gao Y, Lan Y, Liu H, Jiang R (2011). The zinc finger transcription factors Osr1 and Osr2 control synovial joint formation. Dev Biol.

[58] Rankin SA, Gallas AL, Neto A, Gómez-Skarmeta JL, Zorn AM (2012). Suppression of Bmp4 signaling by the zinc-finger repressors Osr1 and Osr2 is required for Wnt/β-catenin-mediated lung specification in Xenopus. Development.

[59] Hong H, Yang L, Stallcup MR (1999). Hormone-independent transcriptional activation and coactivator binding by novel orphan nuclear receptor ERR3. J Biol Chem.

[60] Jeong B-C, Lee Y-S, Park Y-Y, Bae I-H, Kim D-K, Koo S-H, Choi H-R, Kim S-H, Franceschi RT, Koh J-T, Choi H-S (2009). The orphan nuclear receptor estrogen receptor-related receptor gamma negatively regulates BMP2-induced osteoblast differentiation and bone formation. J Biol Chem.

[61] Hay ED, Fischman DA (1961). Origin of the blastema in regenerating limbs of the newt Triturus viridescens. Dev Biol.

[62] Landeira D, Sauer S, Poot R, Dvorkina M, Mazzarella L, Jørgensen HF, Pereira CF, Leleu M, Piccolo FM, Spivakov M, Brookes E, Pombo A, Fisher C, Skarnes WC, Snoek T, Bezstarosti K, Demmers J, Klose RJ, Casanova M, Tavares L, Brockdorff N, Merkenschlager M, Fisher AG (2010). Jarid2 is a PRC2 component in embryonic stem cells required for multi-lineage differentiation and recruitment of PRC1 and RNA Polymerase II to developmental regulators. Nat Cell Biol.

[63] Kaneko S, Bonasio R, Saldaña-Meyer R, Yoshida T, Son J, Nishino K, Umezawa A, Reinberg D (2014). Interactions between JARID2 and noncoding RNAs regulate PRC2 recruitment to chromatin. Mol Cell.

[64] Pjanic M, Schmid CD, Gaussin A, Ambrosini G, Adamcik J, Pjanic P, Plasari G, Kerschgens J, Dietler G, Bucher P, Mermod N (2013). Nuclear Factor I genomic binding associates with chromatin boundaries. BMC Genomics.

[65] Pjanic M, Pjanic P, Schmid C, Ambrosini G, Gaussin A, Plasari G, Mazza C, Bucher P, Mermod N (2011). Nuclear factor I revealed as family of promoter binding transcription activators. BMC Genomics.

[66] Waki H, Nakamura M, Yamauchi T, Wakabayashi K-I, Yu J, Hirose-Yotsuya L, Take K, Sun W, Iwabu M, Okada-Iwabu M, Fujita T, Aoyama T, Tsutsumi S, Ueki K, Kodama T, Sakai J, Aburatani H, Kadowaki T (2011). Global mapping of cell type-specific open chromatin by FAIRE-seq reveals the regulatory role of the NFI family in adipocyte differentiation. PLoS Genet.

[67] Urayama S, Semi K, Sanosaka T, Hori Y, Namihira M, Kohyama J, Takizawa T, Nakashima K (2013). Chromatin Accessibility at a STAT3 Target Site Is Altered Prior to Astrocyte Differentiation. Cell Struct Funct.

[68] Piper M, Barry G, Hawkins J, Mason S, Lindwall C, Little E, Sarkar A, Smith AG, Moldrich RX, Boyle GM, Tole S, Gronostajski RM, Bailey TL, Richards LJ (2010). NFIA controls telencephalic progenitor cell differentiation through repression of the Notch effector Hes1. J Neurosci.

[69] Pickart CM (2001). Mechanisms Underlying Ubiquitination. Annu Rev Biochem.

[70] Goss RJ (1969). Principles of regeneration.

[71] Fischle W, Wang Y, David Allis C (2003). Binary switches and modification cassettes in histone biology and beyond. Nat Cell Biol.

[72] Team, R. Core (2014). R: a language and environment for statistical computing.

[73] Milligan GW, Cooper MC (1985). An examination of procedures for determining the number of clusters in a data set. Psychometrika.

[74] Johnson SC (1967). Hierarchical clustering schemes. Psychometrika.

[75] Onda H, Goldhamer DJ, Tassava RA (1990). An extracellular matrix molecule of newt and axolotl regenerating limb blastemas and embryonic limb buds: immunological relationship of MT1 antigen with tenascin. Development.

[76] Kass M, Witkin A (1987). Analyzing oriented patterns. Comp Vision Graphics, and Image Proc.

[77] Liu Q, Chen J, Gao X, Ding J, Tang Z, Zhang C, Chen J, Li L, Chen P, Wang J (2015). Identification of stage-specific markers during differentiation of hair cells from mouse inner ear stem cells or progenitor cells in vitro. Int J Biochem Cell Biol.

[78] Farias EF, Ong DE, Ghyselinck NB, Nakajo S, Kuppumbatti YS, Mira y Lopez R (2005). Cellular retinol-binding protein I, a regulator of breast epithelial retinoic acid receptor activity, cell differentiation, and tumorigenicity. J Natl Cancer Inst.

[79] Lauing KL, Cortes M, Domowicz MS, Henry JG, Baria AT, Schwartz NB (2014). Aggrecan is required for growth plate cytoarchitecture and differentiation. Dev Biol.

[80] de Crombrugghe B, Lefebvre V, Behringer RR, Bi W, Murakami S, Huang W (2000). Transcriptional mechanisms of chondrocyte differentiation. Matrix Biology.

[81] Sun J, Ishii M, Ting M-C, Maxson R (2013). Foxc1 controls the growth of the murine frontal bone rudiment by direct regulation of a Bmp response threshold of Msx2. Development.

[82] Francis-West PH, Abdelfattah A, Chen P, Allen C, Parish J, Ladher R, Allen S, MacPherson S, Luyten FP, Archer CW (1999). Mechanisms of GDF-5 action during skeletal development. Development.

[83] James CG, Ulici V, Tuckermann J, Underhill TM, Beier F (2007). Expression profiling of Dexamethasone-treated primary chondrocytes identifies targets of glucocorticoid signalling in endochondral bone development. BMC Genomics.

[84] Kessels MY, Huitema LFA, Boeren S, Kranenbarg S, Schulte-Merker S, van Leeuwen JL, de Vries SC (2014). Proteomics analysis of the zebrafish skeletal extracellular matrix. PLoS ONE.

[85] Seo H-S, Serra R (2009). Tgfbr2 is required for development of the skull vault. Dev Biol.

[86] Rudnicki MA, Schnegelsberg P, Stead RH, Braun T (1993). MyoD or Myf-5 is required for the formation of skeletal muscle. Cell.

[87] Shi N, Guo X, Chen S-Y (2014). Olfactomedin 2, a novel regulator for transforming growth factor-β-induced smooth muscle differentiation of human embryonic stem cell-derived mesenchymal cells. Mol Biol Cell.

[88] Havis E, Bonnin M-A, Olivera-Martinez I, Nazaret N, Ruggiu M, Weibel J, Durand C, Guerquin M-J, Bonod-Bidaud C, Ruggiero F, Schweitzer R, Duprez D (2014). Transcriptomic analysis of mouse limb tendon cells during development. Development.

[89] Mata X, Ducasse A, Vaiman A, Diribarne M, Fraud A-S, Guérin G (2010). Genomic structure, polymorphism and expression of ACCN1 and ACCN3 genes in the horse. Anim Genet.

[90] Moore R, Champeval D, Denat L, Tan S-S, Faure F, Julien-Grille S, Larue L (2004). Involvement of cadherins 7 and 20 in mouse embryogenesis and melanocyte transformation. Oncogene.

[91] Lyons PJ, Ma L-H, Baker R, Fricker LD (2010). Carboxypeptidase A6 in zebrafish development and implications for VIth cranial nerve pathfinding. PLoS ONE.

[92] Sasabe J, Miyoshi Y, Suzuki M, Mita M, Konno R, Matsuoka M, Hamase K, Aiso S (2012). D-amino acid oxidase controls motoneuron degeneration through D-serine. Proc Natl Acad Sci.

[93] Akaneya Y, Sohya K, Kitamura A, Kimura F, Washburn C, Zhou R, Ninan I, Tsumoto T, Ziff EB (2010). Ephrin-A5 and EphA5 interaction induces synaptogenesis during early hippocampal development. PLoS ONE.

[94] Ruskamo S, Chukhlieb M, Vahokoski J, Bhargav SP, Liang F, Kursula I, Kursula P (2012). Juxtanodin is an intrinsically disordered F-actin-binding protein. Sci Rep.

[95] Paulsen SJ, Christensen MT, Vrang N, Larsen LK (2008). The putative neuropeptide TAFA5 is expressed in the hypothalamic paraventricular nucleus and is regulated by dehydration. Brain Res.

[96] Matsumata M, Sakayori N, Maekawa M, Owada Y, Yoshikawa T, Osumi N (2012). The effects of Fabp7 and Fabp5 on postnatal hippocampal neurogenesis in the mouse. Stem Cells.

[97] Meyer RC, Giddens MM, Schaefer SA, Hall RA (2013). GPR37 and GPR37L1 are receptors for the neuroprotective and glioprotective factors prosaptide and prosaposin. Proc Natl Acad Sci.

[98] O'Sullivan ML, Martini F, Daake von S, Comoletti D, Ghosh A (2014). LPHN3, a presynaptic adhesion-GPCR implicated in ADHD, regulates the strength of neocortical layer 2/3 synaptic input to layer 5. Neural Dev.

[99] Dai J, Buhusi M, Demyanenko GP, Brennaman LH, Hruska M, Dalva MB, Maness PF (2013). Neuron glia-related cell adhesion molecule (NrCAM) promotes topographic retinocollicular mapping. PLoS ONE.

[100] Decourt B, Hillman D, Bouleau Y, Dulon D, Hafidi A (2009). Is otospiralin inner ear specific? Evidence for its expression in mouse brain. Int J Dev Neurosci.

[101] Ariga H, Takahashi-Niki K, Kato I, Maita H, Niki T, Iguchi-Ariga SMM (2013). Neuroprotective function of DJ-1 in Parkinson’s disease. Oxid Med Cell Longev.

[102] O'Leary C, Cole SJ, Langford M, Hewage J, White A, Cooper HM (2013). RGMa regulates cortical interneuron migration and differentiation. PLoS ONE.

[103] Ma CHE, Brenner GJ, Omura T, Samad OA, Costigan M, Inquimbert P, Niederkofler V, Salie R, Sun CC, Lin HY, Arber S, Coppola G, Woolf CJ, Samad TA (2011). The BMP coreceptor RGMb promotes while the endogenous BMP antagonist noggin reduces neurite outgrowth and peripheral nerve regeneration by modulating BMP signaling. J Neurosci.

[104] Pineda M, Font M, Bassi MT, Manzoni M, Borsani G, Marigo V, Fernández E, Río RMD, Purroy J, Zorzano A, Nunes V, Palacín M (2004). The amino acid transporter asc-1 is not involved in cystinuria. Kidney Int.

[105] Christophe-Hobertus C, Szpirer C, Guyon R, Christophe D (2001). Identification of the gene encoding Brain Cell Membrane Protein 1 (BCMP1), a putative four-transmembrane protein distantly related to the Peripheral Myelin Protein 22/Epithelial Membrane Proteins and the Claudins. BMC Genomics.

[106] Kubota Y, Oike Y, Satoh S, Tabata Y, Niikura Y, Morisada T, Akao M, Urano T, Ito Y, Miyamoto T, Nagai N, Koh GY, Watanabe S, Suda T (2005). Cooperative interaction of Angiopoietin-like proteins 1 and 2 in zebrafish vascular development. Proc Natl Acad Sci U S A.

[107] Takeuchi T, Adachi Y, Nagayama T (2011). Expression of a secretory protein C1qTNF6, a C1qTNF family member, in hepatocellular carcinoma. Anal Cell Pathol (Amst).

[108] Xie J, Yoon J, Yang S-S, Lin S-H, Huang C-L (2013). WNK1 protein kinase regulates embryonic cardiovascular development through the OSR1 signaling cascade. J Biol Chem.

[109] Sun W, Yao L, Jiang B, Shao H, Zhao Y, Wang Q (2012). A role for Cdkl1 in the development of gastric cancer. Acta Oncol.

[110] Jalili A, Wagner C, Pashenkov M, Pathria G, Mertz KD, Widlund HR, Lupien M, Brunet J-P, Golub TR, Stingl G, Fisher DE, Ramaswamy S, Wagner SN (2012). Dual suppression of the cyclin-dependent kinase inhibitors CDKN2C and CDKN1A in human melanoma. J Natl Cancer Inst.

[111] Qi Y, Chang L, Li H, Yu G, Xiao W, Xia D, Guan W, Yang Y, Lang B, Deng K-L, Yao W-M, Ye Z-Q, Zhuang Q-Y (2013). Over-expression of LRIG3 suppresses growth and invasion of bladder cancer cells. J Huazhong Univ Sci Technol Med Sci.

[112] Guo F-J, Zhang W-J, Li Y-L, Liu Y, Li Y-H, Huang J, Wang J-J, Xie P-L, Li G-C (2010). Expression and functional characterization of platelet-derived growth factor receptor-like gene. World J Gastroenterol.

[113] Wang T, Wu H, Li Y, Szulwach KE, Lin L, Li X, Chen I-P, Goldlust IS, Chamberlain SJ, Dodd A, Gong H, Ananiev G, Han JW, Yoon Y-S, Rudd MK, Yu M, Song C-X, He C, Chang Q, Warren ST, Jin P (2013). Subtelomeric hotspots of aberrant 5-hydroxymethylcytosine-mediated epigenetic modifications during reprogramming to pluripotency. Nat Cell Biol.

[114] Dobashi S, Katagiri T, Hirota E, Ashida S, Daigo Y, Shuin T, Fujioka T, Miki T, Nakamura Y (2009). Involvement of TMEM22 overexpression in the growth of renal cell carcinoma cells. Oncol Rep.

[115] Berry R, Harewood L, Pei L, Fisher M, Brownstein D, Ross A, Alaynick WA, Moss J, Hastie ND, Hohenstein P, Davies JA, Evans RM, FitzPatrick DR (2011). Esrrg functions in early branch generation of the ureteric bud and is essential for normal development of the renal papilla. Hum Mol Genet.

[116] Zhang H, Pao LI, Zhou A, Brace AD, Halenbeck R, Hsu AW, Bray TL, Hestir K, Bosch E, Lee E, Wang G, Liu H, Wong BR, Kavanaugh WM, Williams LT (2014). Deorphanization of the human leukocyte tyrosine kinase (LTK) receptor by a signaling screen of the extracellular proteome. Proc Natl Acad Sci.

[117] Sun X, Mariani FV, Martin GR (2002). Functions of FGF signalling from the apical ectodermal ridge in limb development. Nature.

[118] Lemos TA, Passos DO, Nery FC, Kobarg J (2003). Characterization of a new family of proteins that interact with the C-terminal region of the chromatin-remodeling factor CHD-3. FEBS Lett.

[119] Li Y, Dudley AT (2009). Noncanonical frizzled signaling regulates cell polarity of growth plate chondrocytes. Development.

[120] You A, Tong JK, Grozinger CM, Schreiber SL (2001). CoREST is an integral component of the CoREST- human histone deacetylase complex. Proc Natl Acad Sci U S A.

[121] Kohroki J, Nishiyama T, Nakamura T, Masuho Y (2005). ASB proteins interact with Cullin5 and Rbx2 to form E3 ubiquitin ligase complexes. FEBS Lett.

[122] Cubeñas-Potts C, Goeres JD, Matunis MJ (2013). SENP1 and SENP2 affect spatial and temporal control of sumoylation in mitosis. Mol Biol Cell.

[123] Nishiya T, Matsumoto K, Maekawa S, Kajita E, Horinouchi T, Fujimuro M, Ogasawara K, Uehara T, Miwa S (2011). Regulation of inducible nitric-oxide synthase by the SPRY domain- and SOCS box-containing proteins. J Biol Chem.

[124] Walden H, Podgorski MS, Huang DT, Miller DW, Howard RJ, Minor DL, Holton JM, Schulman BA (2003). The structure of the APPBP1-UBA3-NEDD8-ATP complex reveals the basis for selective ubiquitin-like protein activation by an E1. Mol Cell.

[125] Borrelli S, Candi E, Hu B, Dolfini D, Ravo M, Grober OMV, Weisz A, Dotto GP, Melino G, Viganò MA, Mantovani R (2010). The p63 target HBP1 is required for skin differentiation and stratification. Cell Death Differ.

[126] Lee Y-F, Lee H-J, Chang C (2002). Recent advances in the TR2 and TR4 orphan receptors of the nuclear receptor superfamily. J Steroid Biochem Mol Biol.

[127] Bava F-A, Eliscovich C, Ferreira PG, Miñana B, Ben-Dov C, Guigó R, Valcárcel J, Méndez R (2013). CPEB1 coordinates alternative 3'-UTR formation with translational regulation. Nature.

[128] Williams SG, Hall KB (2014). Binding affinity and cooperativity control U2B″/snRNA/U2A' RNP formation. Biochemistry.

[129] Lugtenberg D, Yntema HG, Banning MJG, Oudakker AR, Firth HV, Willatt L, Raynaud M, Kleefstra T, Fryns J-P, Ropers H-H, Chelly J, Moraine C, Gecz J, van Reeuwijk J, Nabuurs SB, de Vries BBA, Hamel BCJ, de Brouwer APM, van Bokhoven H (2006). ZNF674: a new kruppel-associated box-containing zinc-finger gene involved in nonsyndromic X-linked mental retardation. Am J Hum Genet.

[130] Andjelković N, Zolnierowicz S, Van Hoof C, Goris J, Hemmings BA (1996). The catalytic subunit of protein phosphatase 2A associates with the translation termination factor eRF1. EMBO J.

[131] Couté Y, Kindbeiter K, Belin S, Dieckmann R, Duret L, Bezin L, Sanchez J-C, Diaz J-J (2008). ISG20L2, a novel vertebrate nucleolar exoribonuclease involved in ribosome biogenesis. Mol Cell Proteomics.

[132] Chen J, Patton JR (2000). Pseudouridine synthase 3 from mouse modifies the anticodon loop of tRNA. Biochemistry.

[133] Shim KS, Bergelson JM, Furuse M, Ovod V, Krude T, Lubec G (2003). Reduction of chromatin assembly factor 1 p60 and C21orf2 protein, encoded on chromosome 21, in Down syndrome brain. J Neural Transm Suppl.

[134] Ashrafian H, Docherty L, Leo V, Towlson C, Neilan M, Steeples V, Lygate CA, Hough T, Townsend S, Williams D, Wells S, Norris D, Glyn-Jones S, Land J, Barbaric I, Lalanne Z, Denny P, Szumska D, Bhattacharya S, Griffin JL, Hargreaves I, Fernandez-Fuentes N, Cheeseman M, Watkins H, Dear TN (2010). A mutation in the mitochondrial fission gene Dnm1l leads to cardiomyopathy. PLoS Genet.

[135] Handley MT, Mégarbané A, Meynert AM, Brown S, Freyer E, Taylor MS, Jackson IJ, Aligianis IA (2014). Loss of ALDH18A1 function is associated with a cellular lipid droplet phenotype suggesting a link between autosomal recessive cutis laxa type 3A and Warburg Micro syndrome. Mol Genet Genomic Med.

[136] Martinez-Dominguez MT, Justesen J, Kruse TA, Hansen LL (1998). Assignment of the human mitochondrial tryptophanyl-tRNA synthetase (WARS2) to 1p13.3&rarr;p13.1 by radiation hybrid mapping. Cytogenet Genome Res.

[137] Shahni R, Wedatilake Y, Cleary MA, Lindley KJ, Sibson KR, Rahman S (2013). A distinct mitochondrial myopathy, lactic acidosis and sideroblastic anemia (MLASA) phenotype associates with YARS2 mutations. Am J Med Genet A.

[138] Brand F, Schumacher S, Kant S, Menon MB, Simon R, Turgeon B, Britsch S, Meloche S, Gaestel M, Kotlyarov A (2012). The extracellular signal-regulated kinase 3 (mitogen-activated protein kinase 6 [MAPK6])-MAPK-activated protein kinase 5 signaling complex regulates septin function and dendrite morphology. Mol Cell Biol.

[139] Yamada M, Clark J, Iulianella A (2014). MLLT11/AF1q is differentially expressed in maturing neurons during development. Gene Expr Patterns.

[140] Klinger M, Diekmann H, Heinz D, Hirsch C, Hanwehr von SH, Petrausch B, Oertle T, Schwab ME, Stuermer C (2004). Identification of two nogo/rtn4 genes and analysis of Nogo-A expression in Xenopus laevis. Mol Cell Neurosci.

[141] Kang PB, Gooch CL, McDermott MP, Darras BT, Finkel RS, Yang ML, Sproule DM, Chung WK, Kaufmann P, de Vivo DC, Muscle Study Group and the Pediatric Neuromuscular Clinical Research Network for Spinal Muscular Atrophy (2014). The motor neuron response to SMN1 deficiency in spinal muscular atrophy. Muscle Nerve.

