# Supplementary figures and images for: Positional plasticity in regenerating Amybstoma mexicanum limbs is associated with cell proliferation and pathways of cellular differentiation
Source: BMC Dev Biol. 2015 Nov 23;15:45. doi: 10.1186/s12861-015-0095-4 (PMC4657325; doi:10.1186/s12861-015-0095-4)

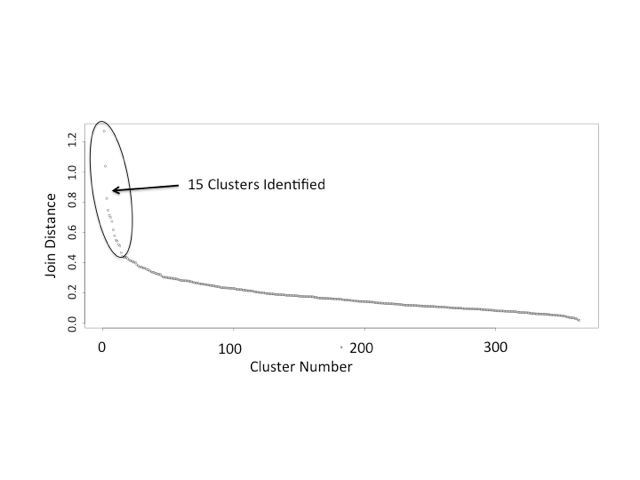

Supplement: Additional file 2: Figure S1. — Selection of 15 clusters of gene expression in EB, apical-LB, and basal-LB in different locations on the P/D limb axis. Join distance (1-Pearson’s correlation, y-axis) plotted as a function of number of clusters. After fifteen clusters, join distances reach an asymptote, suggesting that using more than fifteen groups provides little additional information (small distance indicates separating similarly expressed genes), while using more than fifteen clusters results in joining dissimilar groups (high distance). (TIFF 1203 kb) [file 12861_2015_95_MOESM2_ESM.tiff]

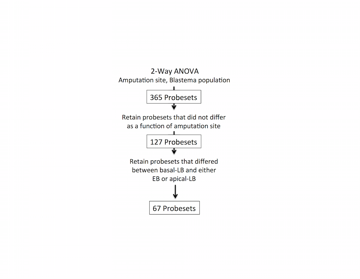

Supplement: Additional file 3: Figure S3. — Diagram showing the statistical strategy used to identify 67 candidate probesets that were expressed differently between EB/apical-LB and basal-LB. We took the 365 significant genes identified using two-way ANOVA and filtered them using t-tests (α = 0.05). First, we identified all probesets (N = 127) that did not show a significant amputation site effect (p > 0.05) between proximal and distal EB samples, or between proximal and distal basal-LB samples. We then identified all probesets (N = 67) that were significant (p < 0.05) when separately comparing EB and apical–LB samples to the basal-LB sample. (TIFF 395 kb) [file 12861_2015_95_MOESM3_ESM.tiff]

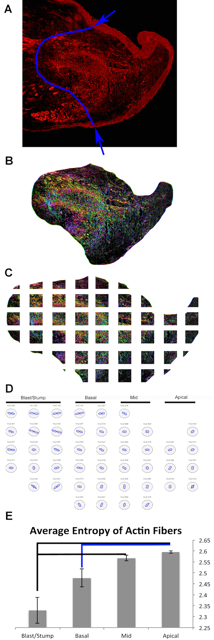

Supplement: Additional file 5: Figure S2. — Workflow of actin quantification on a transverse section of a LB-stage blastema. (A) Confocal image of a transverse section of a LB blastema that had been stained with phalloidin-rhodamin for F-actin (Red). (B) The actin fiber orientations within the blastema mesenchyme of the tissue section was quantified using automated image processing (described in Methods section). Each color represents the orientation of fibers toward a specific direction. For example, red colored fibers are oriented along the proximal/distal axis. (C) The image was divided into small non-overlapping tiles (512 × 512 pixels) each covering approximately 150 square microns of tissue, and all together covered all of the mesenchymal tissue from each section. (D) The discrete entropy of each histogram was computed as a summary statistic to measure the degree of order (alignment) or disorder of actin filaments within the region of tissue spanned by the tile. (E) Histogram representing the average entropy of the tiles from each region of the blastema (Blast/stump, Basal, Mid, Apical, as indicated in (D)). The error bars are the SEM, and Student’s t-test was used to determine statistically signficant changes in organization (p < 0.01). (TIFF 538 kb) [file 12861_2015_95_MOESM5_ESM.tiff]
